# cGAS–STING cytosolic DNA sensing pathway is suppressed by JAK2-STAT3 in tumor cells

**DOI:** 10.1038/s41598-021-86644-x

**Published:** 2021-03-31

**Authors:** Manuel Adrian Suter, Nikki Y. Tan, Chung Hwee Thiam, Muznah Khatoo, Paul A. MacAry, Veronique Angeli, Stephan Gasser, Y. L. Zhang

**Affiliations:** 1 Department of Microbiology, Immunology Programme, National University of Singapore, Singapore, 117456 Singapore; 2 NUS Graduate School for Integrative Sciences and Engineering, National University of Singapore, Singapore, 117597 Singapore

**Keywords:** Cancer microenvironment, Tumour immunology

## Abstract

Deficiencies in DNA repair and DNA degrading nucleases lead to accumulation of cytosolic DNA. cGAS is a critical DNA sensor for the detection of cytosolic DNA and subsequent activation of the STING signaling pathway. Here, we show that the cGAS-STING pathway was unresponsive to STING agonists and failed to induce type I interferon (IFN) expression in many tested human tumor cells including DU145 prostate cancer cells. Inhibition of IL-6 or the downstream JAK2/STAT3 signaling restored responsiveness to STING agonists in DU145 cells. STING activity in murine TRAMP-C2 prostate cancer cells was critical for tumor rejection and immune cell infiltration. Endogenous STING agonists including double-stranded DNA and RNA:DNA hybrids present in TRAMP-C2 cells contribute to tumor rejection, but tumor growth was further suppressed by administration of cGAMP. Intratumoral co-injections of IL-6 significantly reduced the anti-tumor effects of cGAMP. In summary, STING in tumor cells contributes to tumor rejection in prostate cancer cells, but its functions are frequently suppressed in tumor cells in part via JAK2 and STAT3 pathways.

## Introduction

Cyclic GMP-AMP (cGAMP) synthase (cGAS), a cytosolic DNA sensor, catalyzes the synthesis of cyclic dinucleotide cGAMP, which binds and activates Stimulator of interferon (IFN) genes (STING), an endoplasmic reticulum-located adaptor molecule^1, 2^. STING plays a crucial role in mediating inflammation by inducing type I IFN production through recruitment of TANK binding kinase 1 (TBK1) and IFN regulatory factor 3 (IRF3)^3^. Type I IFNs induce the transcription of many anti-viral genes and activate important components of the innate and adaptive immune system including natural killer cells and T cells^4^. *Mb21d1* and *Tmem173*, the genes encoding cGAS and STING respectively, are expressed by many cell types and tissues^5^. cGAS and STING deficiency in various cancer cell lines results in abolished or declined level of type I IFNs in response to exogenous cytosolic DNA, which may contribute to non-inflamed cancer microenvironment^6^.

Early evidence that STING agonists can exert anti-tumor activity was provided by preclinical mouse models using flavone acetic acids and its derivate 5,6-dimethyllxanthenone-4-acetic acid (DMXAA)^7^. Recent studies showed that DMXAA selectively binds mouse, but not human STING, which was suggested to have contributed to its poor performance in phase III trials^8^. The discovery of bacterial cyclic dinucleotides (CDN) and cGAMP allowed the design of STING agonists based on the structure of human STING^9^. Treatment with such novel STING agonists upregulate the expression of pro-inflammatory cytokines and type I IFNs in human and mouse dendritic cells (DCs)^10^. Also, intra-tumoral injection of STING agonists led to complete tumor regressions and protective T-cell responses in several mouse tumor models^7, 11^. Based on these encouraging preclinical data, STING agonists have entered clinical development.

Although the role of STING and STING agonists in immune cells has been well studied, the role of STING in non-immune cells including tumor cells remains poorly understood. We have previously shown that cytosolic DNA in cancer cells induces and maintains low levels of type I IFNs and induce an anti-cancer T-cell response in mouse prostate tumor cells in a STING-dependent manner^12^. Consistently, recent evidence suggests that STING expression in B16 melanoma cells contributes to the activation of immune cells and tumor retardation^13^, suggesting that tumoral STING may play an important role in anti-cancer immunity.

Dysregulated DNA repair processes and expression of nucleases lead to accumulation of cytosolic DNA in tumor cells^12, 14, 15^. Here we show that many tested human cancer cells failed to respond to exogenous STING agonists or double-stranded (ds) DNA. Our data suggest that the cGAMP unresponsiveness of many human tumors is caused by impaired STING activity, but not by dysfunction of pathways downstream of STING. Reduction of cytosolic DNA levels or cGAS deficiency did not restore cGAMP responsiveness of these cancer cells indicating that unresponsiveness was not caused by overstimulation of the pathway. In line with previous studies, we found that responsiveness of tumor cells to STING agonists could be restored by chemical inhibition of IL-6 in DU145 cells or JAK2/STAT3 in all tested cells^16, 17^. STING activity in cancer cells is functionally important as STING expression in prostate TRAMP-C2 cancer cells contributed to their rejection and mediated immune infiltration of the tumor. STING activity was partially mediated by endogenous cGAS agonists including double-stranded DNA and RNA:DNA hybrids present in the cytosol of tumor cells. However, tumor rejection was further boosted by intratumoral injection of cGAMP suggesting that endogenous cGAS agonists fail to fully activate STING in TRAMP-C2 cells. Supporting the finding that JAK2/STAT3 suppresses STING in tumor cells, co-administration of IL-6, a JAK2/STAT3 activator, impaired the anti-tumor effects of cGAMP. In summary, our data show that STING activity in tumor cells contributes to anti-cancer responses, but is often repressed in human cancer cells. Restoration of STING activity by for example blocking JAK2/STAT3 pathways may increase the efficacy of cancer immunotherapies in particular therapies using STING agonists.

## Results

### STING signaling is defective in the majority of tested human cancer cell lines

**Figure 1:**
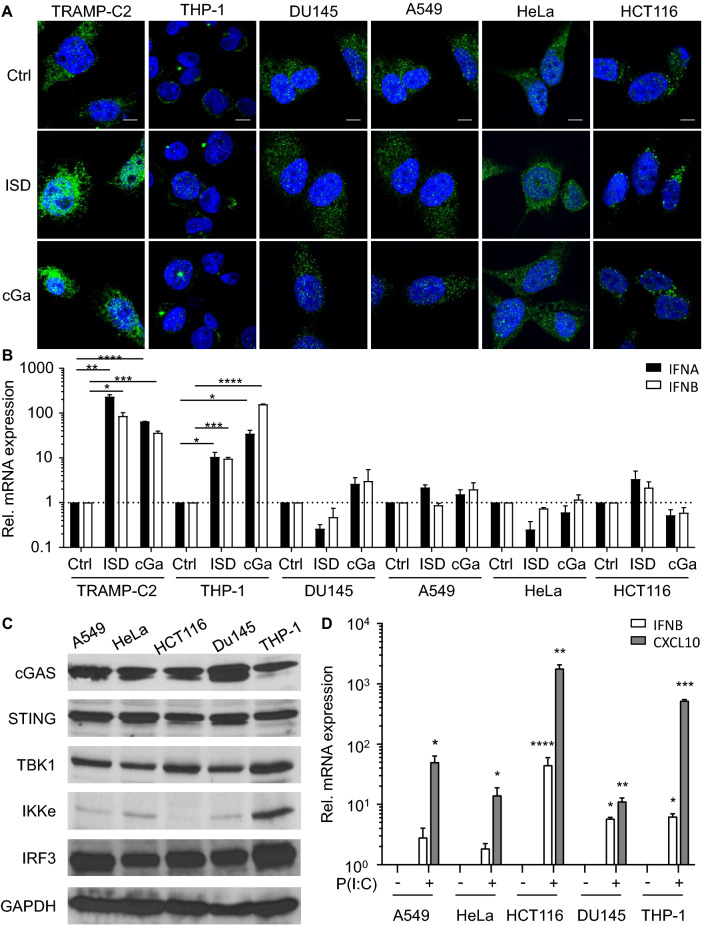
STING Signaling is Defective in the Majority of Tested Human Cancer Cell Lines. (**A**) Murine TRAMP-C2 prostate cancer cells, human THP-1 leukemia cells, human DU145 prostatic carcinoma cells, human A549 lung carcinoma cells, human HeLa cervical adenocarcinoma cells and human HCT116 colorectal carcinoma cells were treated with PBS (ctrl), 2 μg/ml ISD or 2 μg/ml cGAMP (cGa) for 4 h. Endogenous IRF3 expression (green) in treated cells was analyzed by confocal microscopy. Scale bar denotes 10 μm in all images. (**B**) TRAMP-C2, THP-1, DU145, A549, HeLa and HCT116 cells were treated with PBS (ctrl), 2 μg/ml ISD or 2 μg/ml cGAMP (cGa) for 4 h. Treated cells were analyzed for the endogenous expression of *IFNA* (black columns) and *IFNB* (white column) transcripts by real-time PCR. Expression values were normalized to PBS (ctrl)-treated cells. (**C**) Immunoblot analysis of cGAS, STING, TBK1, IKKe, IRF3 and GAPDH levels in A549, HeLa, HCT116, DU145 and THP-1 cells. The cell lysates were equally divided and loaded into different gels. The grouping of blots were cropped from different parts of the same gel, or from different gels. Data are representative of 3 independent experiments. (**D**) A549, HeLa, HCT116, DU145 and THP-1 cells were treated with 25 μM Poly(I:C) for 4 h. Treated cells were analyzed for the expression of *IFNB* (white columns) and *CXCL10* (grey column) transcripts by real-time PCR. Expression values were normalized to mock-treated cells. Data are presented as mean ± SD of 3 independent experiments.

The STING signaling pathway plays a critical role in tumor suppression and immune surveillance^1^. Immune selection of STING expressing cancer cells may lead to loss of STING activity in some tumor cells. In support of this possibility, a recent study found that a majority of human colorectal cancer cells are defective in STING-dependent signaling pathways^18^. In addition, STING was found to be epigenetically silenced in KRAS-LKB1–mutant lung cancers, which may facilitate immune escape^19^. To examine STING activity in different human cancer cells, we first analyzed IRF3 nuclear localization in several human cancer cells upon activation of STING. IRF3 transcriptional activity correlates with nuclear IRF3 translocation, but not with minimal post-translational modifications^20^. cGAMP and ISD induced nuclear translocation of endogenous IRF3 and expression of IRF3 target genes in TRAMP-C2 and THP-1 leukemia cells (Fig. 1A and B). In contrast, endogenous nuclear IRF3 levels or expression of IRF3 target genes did not increase in human DU145 prostatic carcinoma cells, A549 lung carcinoma cells, HeLa cervix carcinoma cells, and HCT116 colorectal carcinoma cells in response to ISD or cGAMP (Fig. 1A and B). Similar findings were observed in cells transduced with a retrovirus encoding an IRF3-GFP fusion protein (Fig. S1A). However, it is worth noting that the steady-state levels of nuclear IRF3-GFP in ISD and cGAMP unresponsive cells were significantly higher when compared to responsive cells. No nuclear localization of the activation-defective IRF3-GFP mutant A7 (IRF3A7-GFP) was observed in all tested cancer cells upon treatments (Fig. S1B).

The inability of the unresponsive cancer cells to respond to STING agonists was unlikely due to mutations in the *Tmem173* or *Mb21d1* genes as nonsynonymous substitutions are not present in either gene in DU145, A549, HeLa and HCT116 cells^21, 22^. The average transcript intensity z-scores for *Tmem173* and *Mb21d1* were within the range found in other cancer cells (n = 60) including ISD/cGAMP responsive cells. While *Tmem173* transcript levels were somewhat lower in A549 cells (z =  − 1.73) and *Mb21d1* transcript levels were decreased in HCT116 cells (z =  − 1.28), no significant difference in STING/cGAS protein levels was observed in either cell line when compared to other tested cells (Fig. 1C and S2). Furthermore, the average transcript intensity z scores for *TBK1* and *IKKe*, two kinases activated by STING, were similar in the analyzed cell lines when compared to other cells (n = 60). The *IKKe* transcript (z =  − 0.9) and protein levels were slightly reduced in HCT116 cells (Fig. 1C and S2)^21, 22^. Finally, ENPP1, which degrades cGAMP was not amplified in any of the tested cells and no gain-of-function mutations were found (Data not shown)^21–23^.

To gain insights into the mechanisms contributing to the inability of these human cancer cells to respond to STING agonists, we treated the different cancer cells with the Toll-like receptor (TLR) 3 agonist Poly(I:C). Similar to cGAMP, Poly(I:C) activates IRF3 through the serine/threonine kinases TBK1 or IKKe^24^. However, unlike the STING-dependent activation of TBK1/IKKe by cGAMP, TLR3 signals require the adaptor TRIF^3^. The TLR3 agonist Poly(I:C) induced the expression of the IRF3 target genes *IFNB* and *CXCL10* in all tested cancer cell lines suggesting that defects upstream of TBK1/IKKe render the cancer cells unresponsive to STING agonists (Fig. 1D). The data also demonstrate that the lower levels of IKKe in HCT116 cells are unlikely to explain their inability to respond to STING agonists. Hence, the inability of some human tumor cells to respond to STING agonist is likely due to the dysfunction of STING activity in these cells.

### Cytosolic DNA does not contribute to STING dysfunction in cancer cells

**Figure 2:**
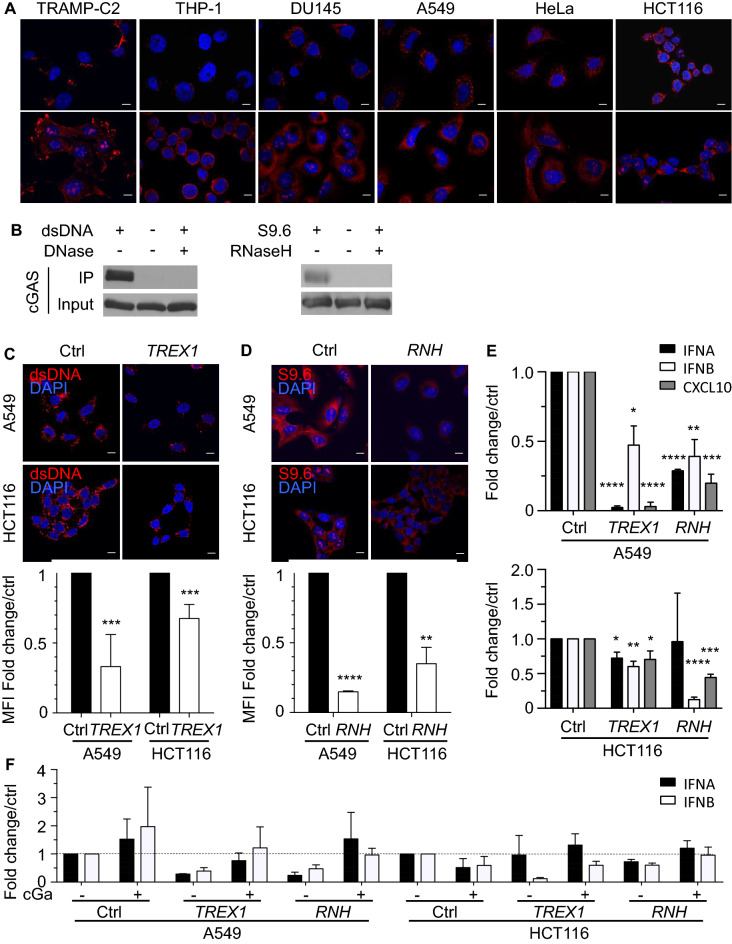
Cytosolic DNA Levels Do Not Contribute to the STING Dysfunction in Human Cancer Cells. (**A**) TRAMP-C2, THP-1, DU145, A549, HeLa, and HCT116 cells were stained for dsDNA or RNA:DNA hybrids recognized by the S9.6 antibody (red) in presence of DAPI (blue). (**B**) Cytosolic dsDNA (left panel) and RNA:DNA hybrids (right panel) in A549 cell lysates were immunoprecipitated with dsDNA-specific or S9.6 antibodies. Precipitates were immunoblotted using a cGAS-specific antibody. A portion of the lysates was treated with 20 units DNase or RNaseH prior to elution. The shown blots are representative of 3 independent experiments. (**C**) A549 or HCT116 cells were transduced with a retrovirus encoding *TREX-1* or control vector and were stained for dsDNA (red) in presence of DAPI (blue). Bar graph shows quantification of the dsDNA MFI ± SD in *TREX1-* (white column) and control- (black column) transduced A549 and HCT116 cells. Values were normalized to the MFI in control cells*.* See also Figure S3. (**D**) *RNASEH1 (RNH)*- or control-transduced A549 and HCT116 cells were stained for RNA:DNA hybrids (red) in the presence of DAPI (blue). Bar graph shows quantification of the RNA:DNA hybrid MFI ± SD in *RNASEH1 (RNH)-* (white column) and control- (black column) transduced A549 and HCT116 cells. Values were normalized to the MFI in control cells*.* See also Figure S3. (**E**) *IFNA* (black column), *IFNB* (white column) and *CXCL10* (grey column) transcript levels in *TREX1-* or *RNASEH1 (RNH)-*transduced A549 and HCT116 cells were determined by real-time PCR. Transcript levels were normalized to transcript levels in control-transduced cells. (**F**) *TREX1-* and *RNASEH1 (RNH)-*transduced A549 and HCT116 cells were treated with PBS (Ctrl) or 2 μg/ml cGAMP (cGa) for 4 h. *IFNA* (black column) and *IFNB* (white column) transcript levels were measured by real-time PCR and normalized to control cells. Scale bar denotes 10 μm in all images. All data are presented as mean ± SD of 3 independent experiments.

Activation of the cytosolic DNA sensor cGAS was found to trigger negative feedback pathways leading to suppression of STING activity^25^. Cytosolic dsDNA and RNA:DNA hybrids were reported to be the major substrates of cGAS^2, 26^. To evaluate whether these DNA species in the cytosol contribute to constitutive cGAS activation and the induction of STING unresponsiveness, we first labelled cGAMP-responsive and unresponsive cancer cell lines for dsDNA and RNA:DNA hybrids in the cytosol. Both dsDNA and RNA:DNA hybrids recognized by the S9.6 antibody were present in the cytosol of all tested tumor cells (Fig. 2A). To investigate if cGAS binds cytosolic DNA in tumor cells, we first co-labelled tumor cells for cGAS and different cytosolic DNA species. Cytosolic dsDNA and RNA:DNA hybrids partially co-localized with cGAS in all tested tumor cells (Figs. S3 and S4).To demonstrate that cGAS physically binds to dsDNA and RNA:DNA hybrids in tumor cells, cytosolic dsDNA and RNA:DNA hybrids were immunoprecipitated in A549 cells. Immunoblot analysis showed that cGAS co-immunoprecipitated with dsDNA and to a lesser degree with RNA:DNA hybrids (Fig. 2B and Fig. S8A). Treatment of the tumor cell lysate with DNase or RNase H abrogated the binding of cGAS to dsDNA or RNA:DNA hybrids, respectively. In summary, our data show that cGAS binds to cytosolic dsDNA and to a lesser degree RNA:DNA hybrids in cancer cells, which may result in the activation of cGAS.

To test if reducing dsDNA or RNA:DNA hybrid levels in unresponsive cells restored STING function, A549 and HCT116 cells were transduced with retroviruses encoding the DNA exonuclease *TREX1* or *RNASEH1*, an endonuclease that degrades the RNA strand in RNA:DNA hybrids (Fig. S5). As expected, the levels of dsDNA decreased in the cytosol of *TREX1-*transduced cells (Fig. 2C), while cytosolic RNA:DNA hybrids levels were lower in cells overexpressing *RNASEH1* (Fig. 2D). The lower levels of cytosolic DNA in *TREX1*- or *RNASEH1-*transduced A549 and HCT116 cells resulted in decreased transcript levels of *IFNA* (*p* < 0.05), *IFNB* (*p* < 0.02), and *CXCL10* (*p* < 0.03) (Fig. 2E). However, overexpression of *TREX1* or *RNASEH1* in A549 and HCT116 cells did not render cells responsive to STING agonists (Fig. 2F), suggesting that cGAMP unresponsiveness of A549 and HCT116 cells was not a result of cytosolic DNA-mediated suppression of STING activity.

### cGAS does not contribute to STING dysfunction

**Figure 3:**
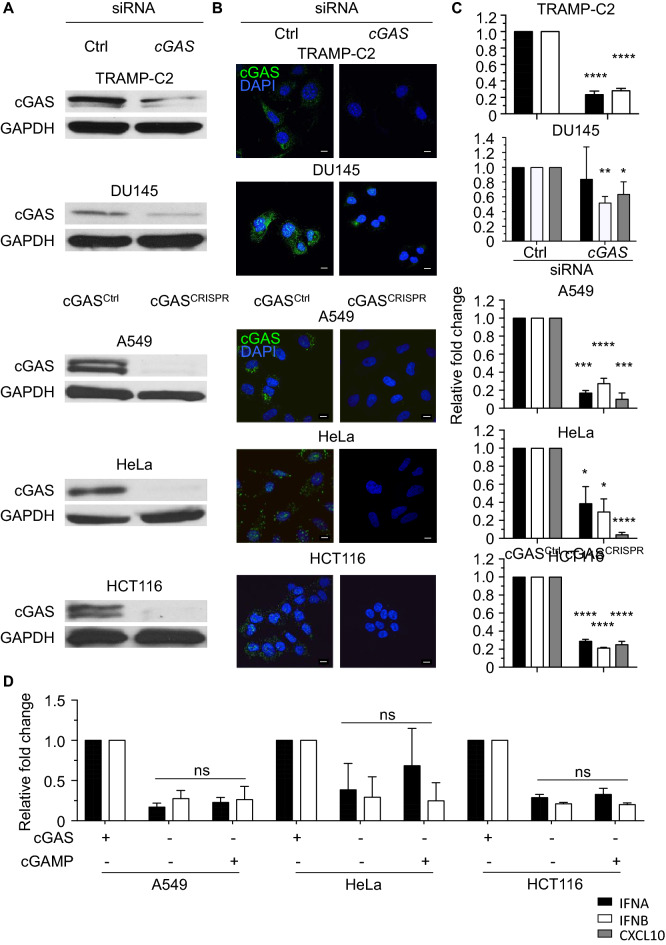
cGAS Does Not Contribute to STING Dysfunction in Human Cancer Cells. (**A**) Immunoblot analysis of cGAS and GAPDH expression in TRAMP-C2 and DU145 cells transfected with control siRNA or *cGAS*-specific siRNA and in cGAS-deficient (cGAS^CRISPR^) or control (cGAS^CTRL^) A549, HeLa, and HCT116 cells. (**B**) cGAS-deficient and wildtype cancer cells were stained for cGAS (green) in the presence of DAPI (blue) and analyzed by confocal microscopy. Scale bars denote 10 μm. (**C**) *IFNA* (black column), *IFNB* (white column) and *CXCL10* (grey column) transcript levels in cGAS-deficient and cGAS expressing TRAMP-C2, DU145, A549, HeLa, and HCT116 cells were determined by real-time PCR. Transcript levels were normalized to levels in the respective control cells. (**D**) cGAS^Ctrl^ and cGAS^CRISPR^ A549, HeLa and HCT116 cells were treated with PBS (−) or 2 μg/ml cGAMP for 4 h followed by measuring *IFNA* (black column) and *IFNB* (white column) transcript levels by real-time PCR. Transcript levels were normalized to levels in untreated cGAS expressing cells. All data represent mean ± SD of 3 independent experiments.

cGAS was functional in TRAMP-C2 and A549 cells as cGAMP was detected in untreated cells and cGAMP levels significantly increased upon transfection of ISD (Fig. S6A). To analyse if cGAS activity contributes to the dysfunction of STING in cGAMP unresponsive cancer cells, we inhibited cGAS expression by *cGAS*-specific siRNAs in DU145 and TRAMP-C2 cells or by a *cGAS*-specific RNA-guided Cas9 nuclease in A549, HeLa, and HCT116 cells^27^. Using different approaches, we confirmed that cGAS expression levels were significantly reduced (*p* < 0.0001) in *cGAS* siRNA-transfected TRAMP-C2 and DU145 cells and that CRISPR-induced frame-shift mutations resulted in cGAS-deficient A549, HeLa, and HCT116 cells (Figs. 3A and B, S6B and C, and S8B). *IFNA*, *IFNB* and *CXCL10* transcript levels were decreased in TRAMP-C2 and DU145 cells transfected with *cGAS* siRNAs and cGAS-deficient A549, HeLa, and HCT116 cells (Fig. 3C) demonstrating that sensing of cytosolic DNA by cGAS contributes to the constitutive expression of type I IFNs in tumor cells. However, cGAS deficiency in cGAMP unresponsive cancer cells did not enable cells to respond to STING agonists (Fig. 3D) showing that STING dysfunction is not maintained by cGAS overstimulation in the tested tumor cells.

### The JAK2/STAT3 pathway contributes to sting dysfunction in tumor cells

**Figure 4:**
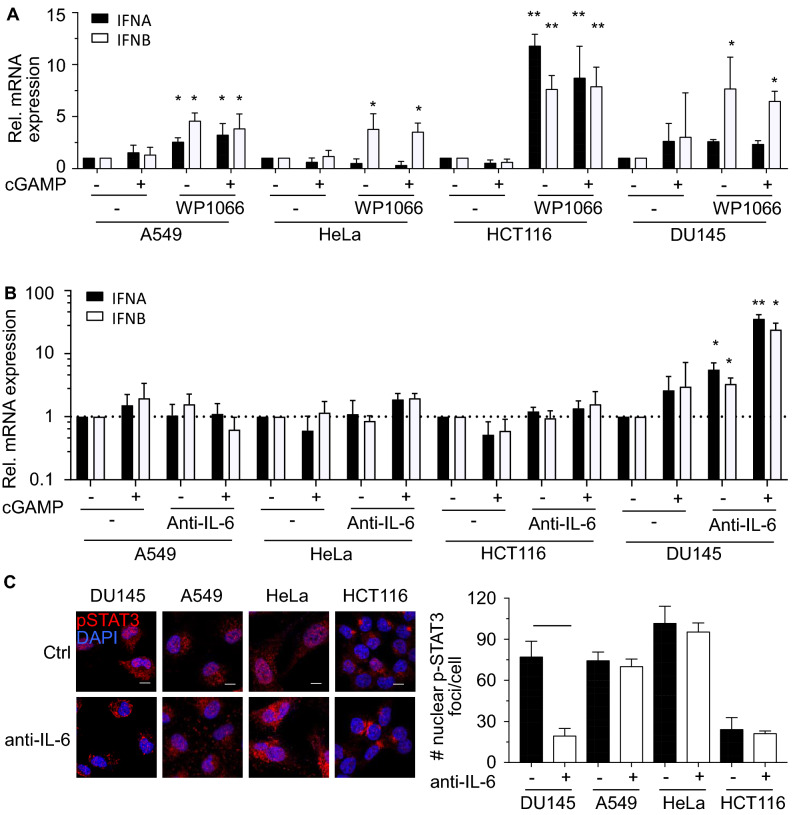
The JAK2/STAT3 Pathway Contributes to STING Dysfunction in Tumor Cells. (**A**) A549, HeLa, HCT116 and DU145 cells were treated with PBS (−), 2 μg/ml cGAMP and/or 7 μM WP1066 for 4 h. Treated cells were analyzed for the expression of *IFNA* (black columns) and *IFNB* (white column) transcripts by real-time PCR. Expression values were normalized to PBS-treated cells. Data are representative of 3 independent experiments. Scale bars denote 10 μm. (**B**) A549, HeLa, HCT116 and DU145 cells were cultured in presence of 1 μg/ml IL-6 neutralizing or isotype control antibodies for 24 h followed by stimulation with 2 μg/ml cGAMP or PBS for 4 h. Treated cells were analyzed for the expression of *IFNA* (black columns) and *IFNB* (white column) transcripts by real-time PCR. Expression values were normalized to PBS-treated cells. (**C**) Representative confocal microscopy images of DU145, A549, HeLa and HCT116 cells stained for phosphorylated STAT3 (red) in the presence of DAPI (blue). Cells were cultured in the presence of 1 μg/ml IL-6 neutralizing or isotype control antibodies. Bar graph below shows quantification of the number of nuclear phosphorylated STAT3 foci in control and treated cancer cells.

Recent reports showed that IL-6 and JAK2/STAT3 pathway negatively regulates STING activity in THP-1 cells^16^ and fibroblastic MRC-5 cells^17^. To explore the role of JAK2/STAT3 in the suppression of type I IFN expression, cells were treated with the JAK2/STAT3 inhibitor WP1066 at doses 3 times above published IC_50_ (Fig. 4A)^28^. Strikingly, inhibition of JAK2/STAT3 activity increased the expression of *IFNA* or *IFNB* transcripts in all tested cell lines. To investigate if cytosolic DNA activates the JAK2/STAT3 pathway via IL-6, we blocked IL-6 signals by using neutralizing antibodies against IL-6. Blocking of IL-6 rendered DU145 cells responsive to cGAMP (Fig. 4B). Blocking of IL-6 correlated with reduced numbers of phosphorylated STAT3 foci in the nucleus of DU145 cells (Fig. 4C). These data suggested that STAT3 is activated by IL-6 in DU145 cells but not the other tested cells. In summary, our data show that activation of the JAK2/STAT3 pathway, which can be activated by diverse signals including IL-6^29^, abrogates the response of cells to endogenous type I IFN inducers such as cGAMP.

### STING function in tumor cells contributes to control of tumor growth and inflammation of the tumor microenvironment

**Figure 5:**
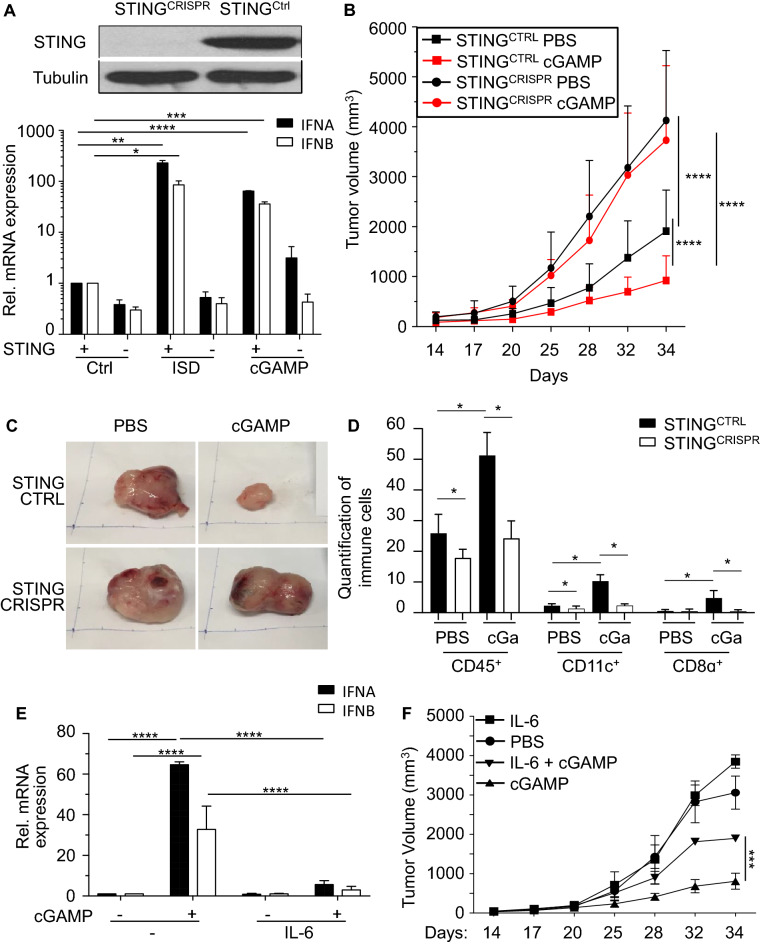
STING Activation in Tumor Cells Contributes to Control of Tumor Growth and Inflammation of Tumor Microenvironment. (**A**) Immunoblot analysis of murine STING^CTRL^ and STING^CRISPR^ TRAMP-C2 prostate cancer cells probed with antibodies for STING and β-tubulin. STING^CTRL^ and STING^CRISPR^ TRAMP-C2 cells were treated with PBS (ctrl), 2 μg/ml ISD or 2 μg/ml cGAMP for 4 h. Treated cells were analyzed for the expression of *Ifna* (black columns) and *Ifnb* (white column) transcripts by real-time PCR. Expression values were normalized to PBS (ctrl)-treated cells. (**B**) Male C57BL/6 mice (n = 10 per group) were subcutaneously injected with 5 × 10^6^ STING expressing (STING^CTRL^; squares) or STING-deficient (STING^CRISPR^; circles) TRAMP-C2 cells. Mice received intra-tumoral injections of (**B**) PBS (black symbols) or (**C**) 25 μg cGAMP (red symbols) on day 14, 17, 20, 25, 28, 32, 34 post-injection (p.i.) of TRAMP-C2 cells. Tumor volumes were measured at indicated time points. Three independent experiments were performed and data are shown as mean ± SD. (**C**) Representative images of subcutaneous prostate tumors removed on day 34 p.i. (**D**) STING expressing (black columns) and STING-deficient (white columns) TRAMP-C2 tumors shown in (**C**) were stained and quantified for the percentage of CD45^+^, CD11c^+^ or CD8α^+^ cells among all DAPI positive cells per section (10 sections per staining, n ≥ 1000 cells analyzed). Data are presented as mean ± SD of 3 independent experiments. (**E**) TRAMP-C2 cells were cultured in presence of 0.1 μg/ml IL-6 recombinant protein for 24 h followed by treatment of 2 μg/ml cGAMP or PBS (−) for 4 h. Treated cells were analyzed for the expression of *IFNA* (black columns) and *IFNB* (white column) transcripts by real-time PCR. Expression values were normalized to PBS (ctrl)-treated cells. (**F**) Male C57BL/6 mice (n = 3 per group) were subcutaneously injected with 7 × 10^6^ TRAMP-C2 cells. Mice were treated with intra-tumoral injections of either PBS (circle), 75 ng murine IL-6 (square), 25 μg cGAMP (triangles) or 75 ng murine IL-6 in combination with 25 μg cGAMP (inverted triangles) on days 14, 17, 20, 25, 28, 32, 34 post-injection (p.i.) of tumor cells. Tumor volumes were measured at indicated time points. Data are representative of 3 independent experiments.

Recent reports have shown significant anti-cancer effects of STING agonists in several mouse cancer models and various small-molecule STING agonists are currently being evaluated in clinical trials^7, 30^. To address if STING activity in tumor cells contributes to the anti-cancer responses, we challenged C57BL/6 mice with STING-sufficient or STING-deficient TRAMP-C2 cells. As expected, STING-deficient TRAMP-C2 cells no longer upregulated type I IFNs levels in response to the STING agonists cGAMP or the cGAS agonist IFN stimulatory DNA (ISD) (Fig. 5A and Fig. S8C). Tumor cells that lacked STING grew significantly faster when compared to STING expressing TRAMP-C2 cells (*p* < 0.001; Fig. 5B). Intratumoral injection of cGAMP 14 days after administration of TRAMP-C2 cells further slowed the growth rate of STING sufficient TRAMP-C2 tumors (*p* < 0.001; Fig. 5B). Strikingly, intratumoral administration of cGAMP had no effect on the growth rate of TRAMP-C2 tumors that were deficient in STING (Fig. 5B and C) suggesting that activation of STING in TRAMP-C2 cells is critical for anti-cancer responses of cGAMP in this model.

STING agonists were shown to mediate tumor infiltration of lymphocytes by inducing the production of pro-inflammatory cytokines and chemokines^31^. Tumor-infiltrating lymphocytes are associated with suppressed tumor growth and favorable prognostic value in patients with different types of cancers^32, 33^. To determine the role of tumoral STING expression in mediating immune cell infiltration of tumors, we stained STING^CTRL^ and STING^CRISPR^ TRAMP-C2 tumors for the presence of different immune cells upon administration of cGAMP. Fewer immune cells expressing the pan-leukocyte marker CD45 were present in STING-deficient TRAMP-C2 tumors when compared to tumors expressing STING (*p* < 0.029; Fig. 5D). Treatment of tumors with cGAMP increased the presence of CD45^+^ immune cells in the tumor microenvironment in a STING-dependent manner (*p* < 0.021; Fig. 5D and Fig. S7A). STING-sufficient TRAMP-C2 tumors contained higher numbers of CD11c^+^ dendritic cells (DCs), which play an important role in anti-cancer immune responses (*p* < 0.03; Fig. 5D and Fig. S7A)^34^. Consistent with the conclusion that STING activation attracts DCs to the tumor microenvironment, intratumoral injection of cGAMP increased the number of CD11c^+^ DCs in the tumor (*p* < 0.02; Fig. 5D and Fig. S7A). CD11c^+^ DCs in tumors did not co-express CD68, a marker for monocytes and macrophages (Fig. S7B). To investigate if STING activation also attracts cytotoxic T cells, tumors were stained for the presence of CD8α^+^ T cells. CD8α^+^ T cells were only rarely observed in the tumor microenvironment of STING-sufficient or -deficient TRAMP-C2 tumors (Figs. 5D and S7A). Intratumoral injection of cGAMP notably increased the number of CD8α^+^ T cells in STING-sufficient tumors, but not in STING-deficient tumors (*p* < 0.05; Fig. 5D). In summary, our data support the conclusion that activation of STING expression in TRAMP-C2 cells contributes to anti-cancer immunity by attracting DCs and T cells.

Our in vitro data suggest that the IL-6 and JAK2/STAT3 pathways suppress STING function in cancer cells. Addition of exogenous IL-6 to TRAMP-C2 cells inhibited their ability to induce the expression of type I IFNs in response to cGAMP (Fig. 5E). To test the ability of IL-6 to suppress the anti-cancer effects of cGAMP in vivo, IL-6 was co-administrated with cGAMP in the TRAMP-C2 prostate cancer model. Co-injection of mouse IL-6 significantly decreased the anti-tumor effects of cGAMP, while IL-6 on its own had no impact on tumor growth, demonstrating that tumor growth promoting effects of the co-injection depended on cGAMP (Fig. 5F). In summary, our data suggest that signals leading to STAT3 activation may counter the effects of STING agonists. Combination of STAT3 small molecule antagonists that are currently being developed or IL-6 inhibitors might be interesting combination partners for STING agonists in future clinical trials.

## Discussion

It was suggested that STING is activated by tumor-derived DNA upon engulfment of necrotic tumor cells by DCs^35^. The subsequent production of type I IFNs and other factors may facilitate cross-presentation of tumor antigens by DCs and activation of tumor-specific T cells. Here we show that the cytosolic DNA accumulates in tumor cells and activates the cGAS/STING pathway. Recognition of cytosolic DNA by this intracellular DNA sensor pathway slows the formation of TRAMP-C2 prostate cancer cells consistent with findings in the B16 melanoma model^13^. The anti-cancer effects of cytosolic DNA may in part be mediated by STING-dependent signals that induce tumor infiltration of immune cells. Interestingly, very few CD8^+^ T cells were present in TRAMP-C2 tumor tissue suggesting that endogenous STING activity in TRAMP-C2 tumors is not sufficient to attract T cells possibly due to an immunosuppressive microenvironment in TRAMP-C2 tumors, which prevents infiltration of T cells^36, 37^.

The anti-cancer effects of STING have led to the evaluation of therapeutic potential of STING agonists, which were effective in several mouse tumor models^7^. Encouragingly, injection of cGAMP was able to enhance T cell infiltration of TRAMP-C2 tumors. While STING activation in antigen-presenting cells (APCs) plays an important role in the anti-cancer effects of STING agonists^38^, STING activation by cGAMP in TRAMP-C2 cells was critical for CD8^+^ T-cell infiltration into the tumor microenvironment. Analogous to stimulation of STING in APCs, cGAMP induced the expression of type I IFNs, pro-inflammatory cytokines and chemokines in TRAMP-C2 cells. The T cell attracting factors released by TRAMP-C2 in response to cGAMP are currently not known, but possibly include chemokines such as CCL5 and CXCL10. Hence, our data indicate that the ability of STING agonists to convert an “excluded infiltrate phenotype” into an “inflamed” tumor depends on STING expression in tumor cells and APCs^39^. Immune cell infiltration of human tumors has been associated with responses to checkpoint inhibitors and favorable clinical outcome in many different tumor types including prostate cancer^40^. High densities of memory CD8^+^ T cells correlate with longer disease-free survival and overall survival. In agreement, infiltration of CD8^+^ T cell and other immune cell in TRAMP-C2 tumors negatively correlated with tumor growth consistent with the conclusion that STING activity in tumor cells is paramount to mount effective anti-cancer immune responses.

We previously found that repair of genomic DNA contributes to the accumulation of cytosolic DNA in prostate cancer cells^12^. Here we show that cytosolic DNA present in many tumor cells, but not normal healthy cells, activates the cGAS/STING pathway. In accordance, cGAS deficiency or overexpression of *RNASEH1* or the nuclease *TREX1* reduced the expression of type I IFN and other IRF3-target genes. However, transfection of exogenous DNA or cGAMP stimulation failed to upregulate the production of type I IFNs in many of tested tumors suggesting that cGAS-STING pathway is suppressed in these tumor cells in line with previous reports^18, 19^. Here we found that unresponsive cells were able to respond to stimuli that activate pathways downstream of STING indicating that the suppression acts at the level of STING and potentially also cGAS. The suppression was not a consequence of cGAS or STING mutations or significant differences in the expression of cGAS or STING. It is also unlikely that cytosolic DNA or constitutive cGAS signals were maintaining the suppression as lowering of cytosolic DNA levels, cGAS deficiency or blocking of type I IFNs (data not shown) failed to restore cGAMP responsiveness of STING. In the prostate cancer cells DU145, blocking of autocrine IL-6 restored the ability of cells to induce type IFN expression in response to cGAMP. In accordance, addition of exogenous IL-6 suppressed cGAMP-induced type I IFN expression and anti-tumor responses. It is possible that other IL-6-like signals that activate the JAK2/STAT3 pathway are mediating some of the suppression in other cancer cells as treatment of cGAMP-unresponsive cancer cells with the JAK2/STAT3 inhibitor WP1066 upregulated type I IFN expression in the tested cancer cells.

In summary, we found that many human cancer cell lines fail to respond to STING agonists, which can be rescued by JAK2/STAT3 inhibitors in some cases. Recent reports also found evidence for STING silencing in human cancer samples^19^. Future studies will be needed to investigate the role of the JAK2/STAT3 pathway in silencing STING function in human cancers. Such studies will be of particular importance as STING agonists and STAT3 inhibitors are currently being developed as new anti-cancer therapeutics. IL-6 antagonists such as Tocilizumab and Sarilumab, are approved for rheumatoid arthritis. Our data show that patient stratification based on STING activity in tumors should be considered when recruiting patients for clinical trials. Furthermore, combination of STING agonists with IL-6 antagonists or STAT3 inhibitors may increase the efficacy of STING agonists. Overall, a better understanding of the molecular mechanisms contributing to the unresponsiveness of tumor cells to STING agonists may open new approaches for cancer treatment.

## Methods

Study in this article was approved by the Ethics Committee Institutional Animal Care and Use Committee (IACUC) of the National University of Singapore (NUS) in accordance with the guideline R14-0204. The study on animals was carried out in compliance with the ARRIVE guidelines.

### Mice

4 to 5-week-old male C57BL/6 wild-type mice were purchased from InVivos (Singapore). Mice were housed according to the IACUC guidelines of the National University of Singapore (R14-0204).

5 × 10^6^ cells STING^Ctrl^ or STING^CRISPR^ TRAMP-C2 tumor cells^41^ were inoculated subcutaneously in the left flank of mice. 14 days after administration of tumor cells, mice were treated by intra-tumoral (i.t.) injections of cGAMP (25 µg/mice in 4 µl PBS) on day 14, 17, 20, 25, 28, 32 and 34. Equal volumes of PBS were administrated as control. For IL-6 experiments, 7 × 10^6^ TRAMP-C2 tumor cells were inoculated subcutaneously in the right flank of mice. 14 days post-administration of tumor cells, mice were treated i.t. using cGAMP (25 µg/mice in 10 µl PBS), murine IL-6 (75 ng/mouse in 10 µl PBS), combination (25 µg cGAMP and 75 ng IL-6/mouse in 10 µl PBS) or PBS only as control on day 14, 17, 20, 25, 28 and 32. Measurements of tumors were performed on day 14, 17, 20, 25, 28, 32 and 34 using digital callipers, and the tumor volume was calculated with the formula V = (length × width^2^)/2 ^42^.

### Cell lines

A549, HeLa and THP-1 cells were purchased from ATCC (USA). DU145 was a generous gift by Dr. R.E. Chee (SIgN, Singapore). HCT116 cell line was a gift from Dr. K. Miyagawa (University of Tokyo, Japan). TRAMP-C2 was kindly provided by Dr. D. H. Raulet (University of California, Berkeley). TRAMP-C2 cells were derived from the transgenic adenocarcinoma mouse prostate (TRAMP) model which expresses SV40 large T-antigen in prostate epithelial cells^43^. Cells were cultured either in DMEM/McCoy (Invitrogen) supplemented with 10% heat-inactivated fetal calf serum (FCS, Hyclone, USA) and 1% pen/strep (Invitrogen) or RPMI (Invitrogen) supplemented with 10% FCS and 50 mM 2-mercaptoethanol (Invitrogen)^12^. All cells were grown at 37 °C in a humidified 5% CO_2_ incubator (Thermo Fisher).

### Reagents and constructs

The mouse *cGAS*-specific siRNA (#LQ-055608-01-0002) and human *cGAS*-specific siRNA (#L-015607-02-0005) were purchased from GE Dharmacon (USA). ISD Naked (#tlrl-isdn, 1 μg/μl stock), cGAMP (#tlrl-nacga23, 1 μg/μl stock), and Poly(I:C) (#tlrl-pic, 25 mM stock) were purchased from InvivoGen (Singapore) and dissolved in endotoxin-free water. Mouse IL-6 (#ab198572, 0.1 μg/ul stock) was purchased from Abcam (Singapore). The anti-human IL-6 neutralising antibody (#ab9324, 1 μg/ul stock) was purchased from Abcam (Singapore). The JAK/STAT3 pathway Inhibitor WP1066 (#sc-203282, 20 mM stock) was purchased from Santa Cruz Biotechnology (Singapore) and dissolved in DMSO (Sigma-Aldrich, Singapore). Cells were treated with 25 μM Poly(I:C), 7 μM WP1066, 2 μg/ml cGAMP, ISD or RNA:DNA hybrids for 4 h.

### CRISPR/CAS9

*cGAS* U6-gRNA vector targeting human *cGAS* exon 1 (GGCCCCCATTCTCGTACGGAGGG) was synthesized as described previously^12^. cGAS deficient cells were generated as described in^12^.

### Immunofluorescence and immunohistochemistry

dsDNA and S9.6 stainings were performed as described^12^. cGAS (MB21D1, Sigma-Aldrich) and p-STAT3 (clone D3A7, Cell Signaling Technology, USA) stainings used the same protocol as S9.6. Briefly, cells were fixed and stained with dsDNA (MAB1293, Millipore) or S9.6 DNA:RNA hybrids antibodies according to manufacturer’s instructions followed by anti-mouse IgG-Cy3 (Millipore) antibodies. The RNA:DNA hybrid-specific antibody S9.6 was a kind gift of Dr. D. Koshland (University of California, Berkeley)^44^.

Fresh tumors were snap-frozen in Tissue-Tek OCT Compound (Sakura, Singapore) and 7 µm sections were prepared and fixed in 100% ice cold acetone (Sigma-Aldrich) for 5 min. Sections were blocked with 0.2% BSA in PBS for 10 min at room temperature. Endogenous Biotin-Blocking Kit (#E21390, Thermo Fisher) was used according to the manufacturer’s instructions to block endogenous biotin for CD45-biotin antibody. Primary antibodies used included anti-mouse CD45 biotin (A20, eBioscience), anti-mouse CD68 (FA-11, Bio-Rad), anti-mouse CD11c (HL3, BD Pharmingen), anti-mouse CD8α (4SM15, eBioscience) or corresponding isotype control antibodies (eBioscience). Secondary antibodies (Jackson ImmunoResearch, USA), including anti-rat IgG-Alexa488, streptavidin-Cy3 and anti-armenian hamster IgG-Cy3 were used for detection. Stained sections were stained with 0.5 μg/ml DAPI (#71-03-01, KPL Inc, USA) before mounting with Dako fluorescent mounting medium (Dako, UK).

Metamorph was used for the quantification of mean fluorescence intensities (MFI) and determination of percentage of positive immune cells (Metamorph NX, Molecular Devices, USA).

### Western blotting

Immunoblots were performed as previously described^26^.

### Real-time PCR

RNA extraction and reverse transcription were previously described^12^. The PCR conditions were used according to TaqMan Gene Expression Assays (Thermo Fisher). Triplicates were performed for the PCR reaction using the ABI PRISM 7700 Sequence Detection System (Applied Biosystems, Singapore). Finally, gene-specific values were normalized to the *GAPDH* or *HPRT* levels. Samples prepared without RNA served as negative controls.

### ELISA

For cGAMP measurements, 1 × 10^6^ TRAMP-C2 and A549 cells were grown in a culture dish and treated with 4 μg/ml ISD or water as control for 18 h. Cells were collected and lysed using M-PER mammalian protein extraction reagent. After lysed cells were spun down, supernatant was collected and cGAMP levels were measured using the 2′3′-cGAMP ELISA kit according to manufacturer’s instructions (Cayman Chemical, USA).

### Statistical analysis

Data distribution was determined by Shapiro–Wilk normality test. *P* values were determined using Student’s t-tests, ANOVA or Pearson correlation coefficient analysis as appropriate (Prism 7d, Graphpad). *p < 0.05; **p < 0.01; ***p < 0.005; ****p < 0.001.

## Supplementary Information

Supplementary Information
