# Supplementary material for: cGAS–STING cytosolic DNA sensing pathway is suppressed by JAK2-STAT3 in tumor cells
Source: Sci Rep. 2021 Mar 31;11:7243. doi: 10.1038/s41598-021-86644-x (PMC8012641; doi:10.1038/s41598-021-86644-x)
Supplement: Supplementary file 1 — Supplementary Information [file 41598_2021_86644_MOESM1_ESM.pdf]

cGAS–STING Cytosolic DNA Sensing Pathway is Suppressed by JAK2-STAT3 in Tumor Cells

Manuel Adrian Suter[1], Nikki Y. Tan[1], Thiam Chung Hwee[1], Muznah Khatoo[1], Paul A. MacAry[1], Veronique Angeli[1], Stephan Gasser[1, 2], Y.L. Zhang[1,3,*]

[1]Immunology Programme, Department of Microbiology, National University of Singapore 117456, Singapore

[2]NUS Graduate School for Integrative Sciences and Engineering, National University of Singapore, 117597 Singapore

[3]Roche Innovation Center Shanghai, Shanghai 201203, China

[*]Corresponding author:

Y.L. Zhang, Roche Innovation Center Shanghai, Shanghai, 201203, China; E-mail: 414700477@qq.com

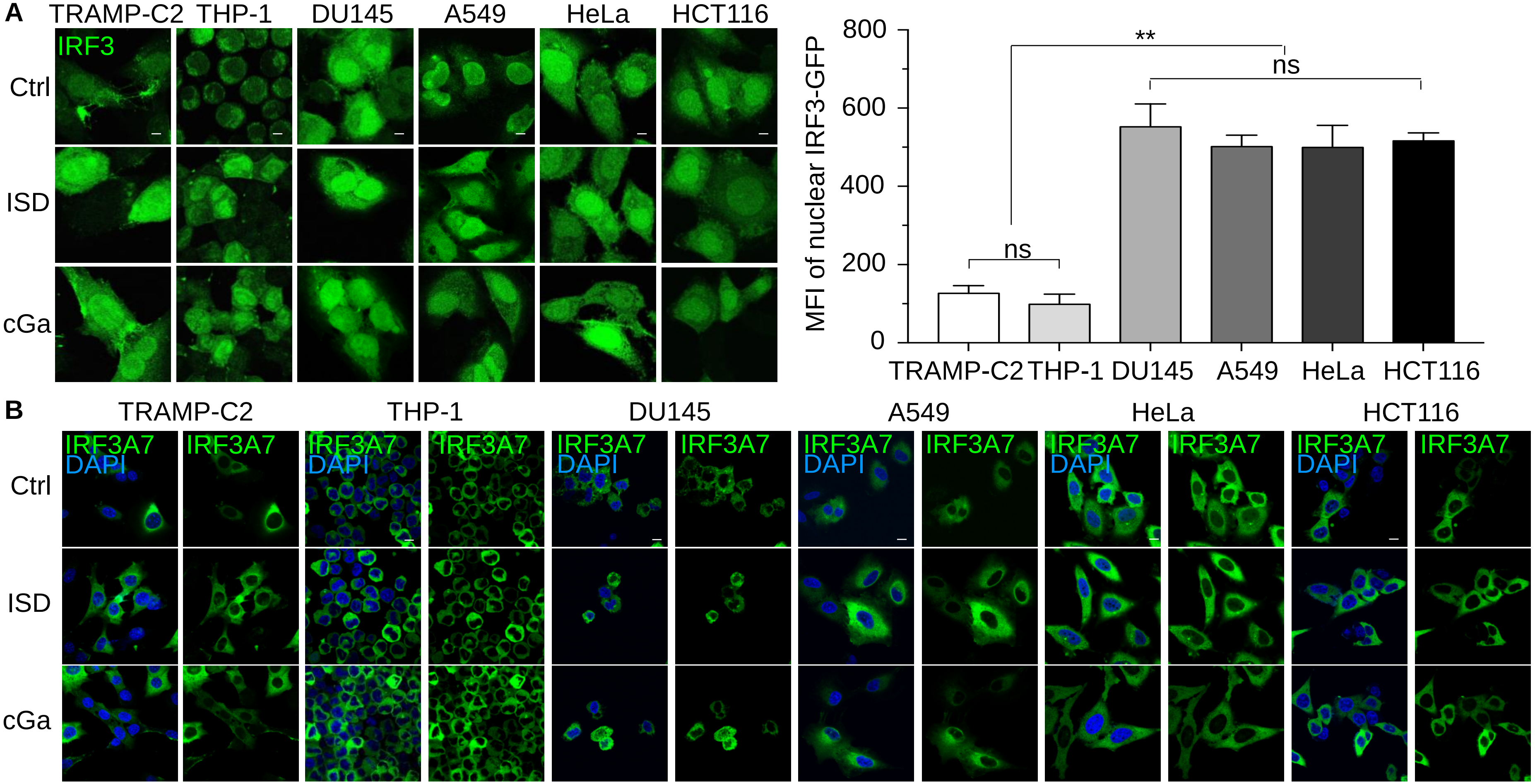

Figure S1. Analysis of IRF3-GFP and IRF3A7-GFP Expression in Tumor Cells.

A, Indicated cells were transduced with a retrovirus encoding an IRF3-GFP fusion protein. Transduced cells were treated with PBS (ctrl), 2 μg/ml ISD or 2 μg/ml cGAMP (cGa) for 4 hours. GFP expression (green) in treated cells was analyzed by confocal microscopy. Bar graph shows quantification of the MFI ± SD of nuclear IRF3-GFP in control cells. **B**, Indicated cells were transduced with a retrovirus encoding an IRF3A7-GFP fusion protein. Transduced cells were treated with PBS (ctrl), 4 μg ISD or 4 μg cGAMP for 4 hours. GFP expression (green) in treated cells was analyzed by confocal microscopy in presence of DAPI (blue). All data are representative of 3 independent experiments. Scale bar denotes 10 μm in all images.

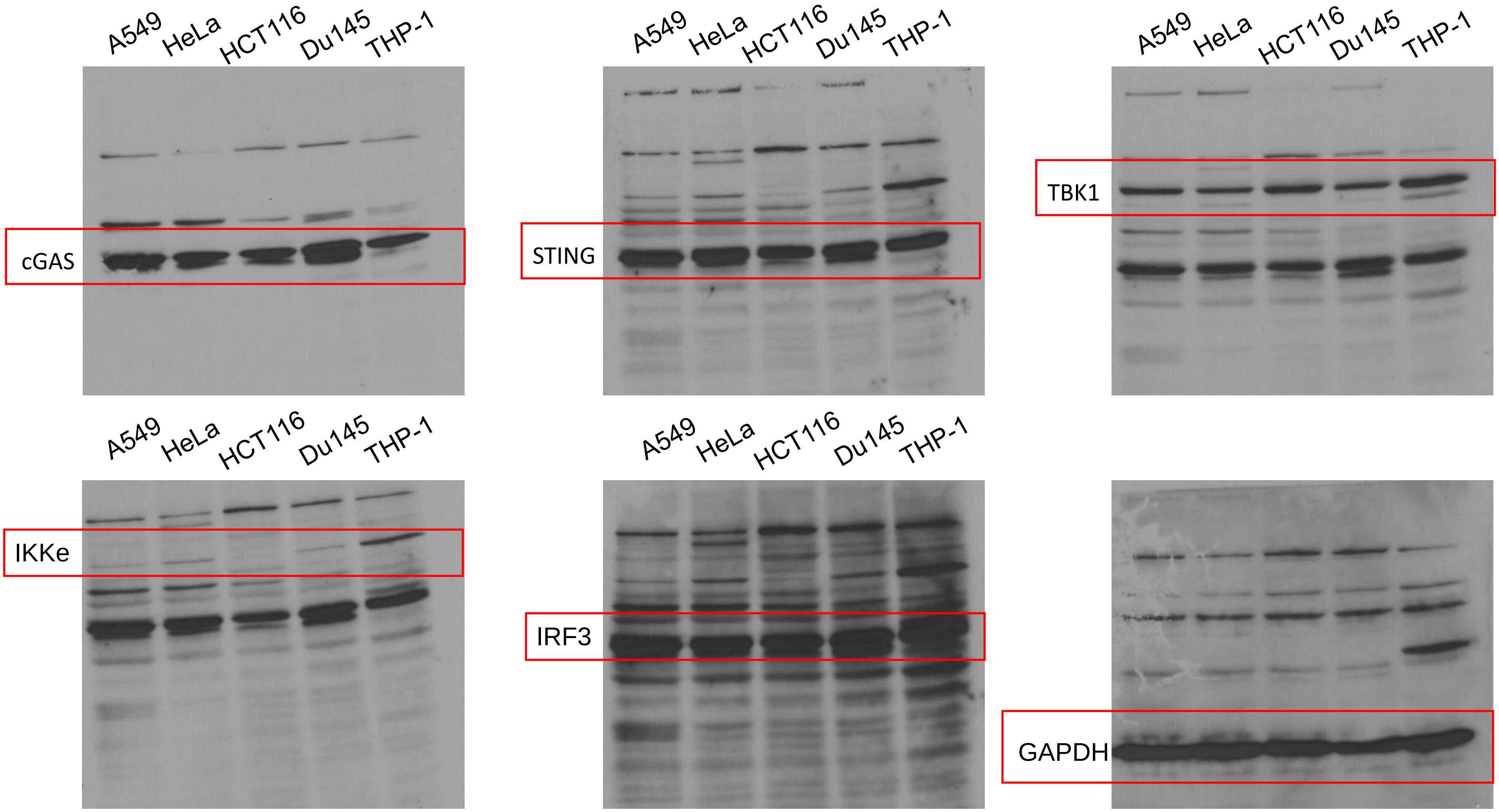

Figure S2. Full images of western blots for Figure 1C.

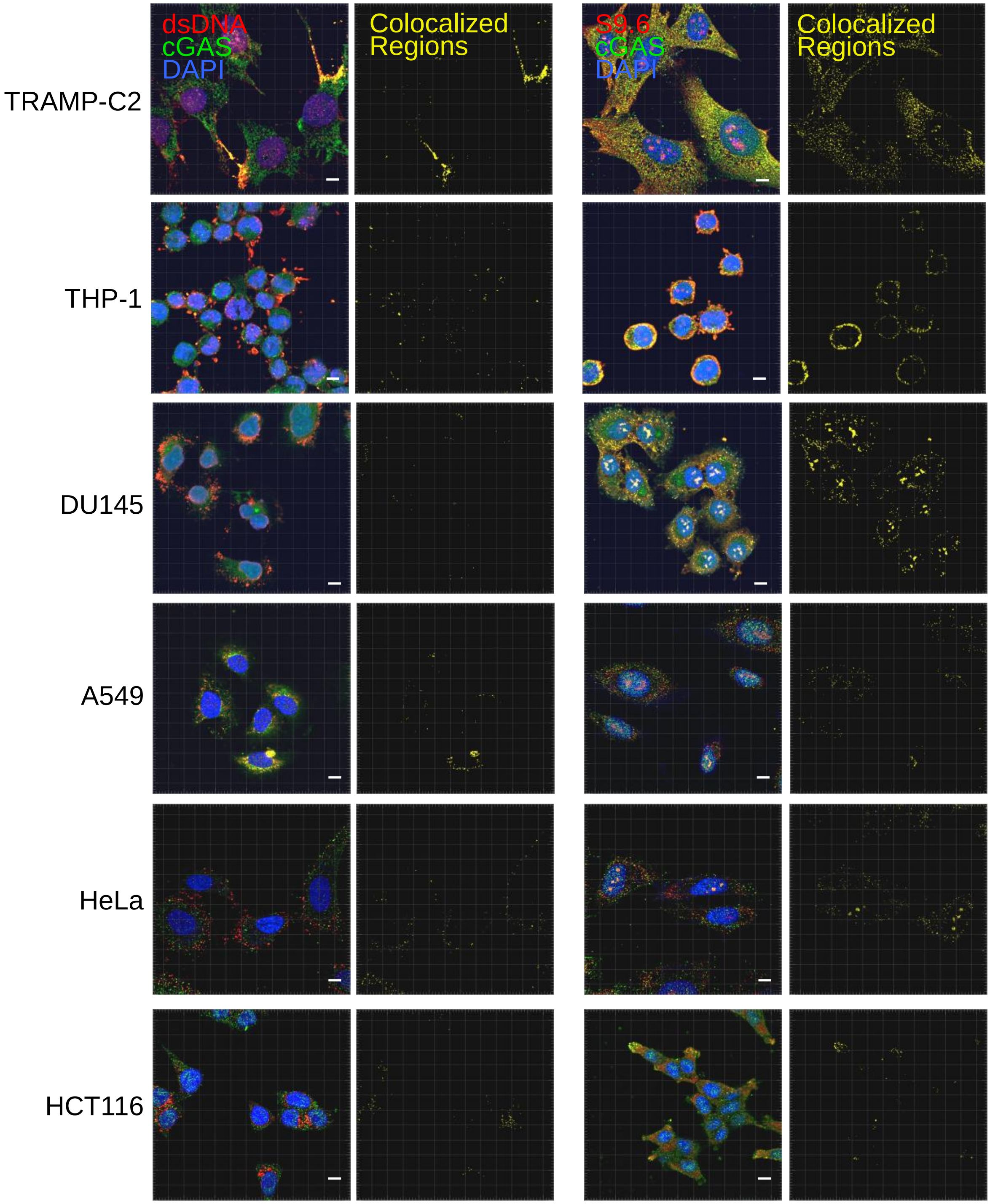

Figure S3. Co-staining of Cytosolic DNA and cGAS in Tumor Cells.

TRAMP-C2, THP-1, DU145, A549, HeLa, and HCT116 cells were co-stained for dsDNA or RNA:DNA hybrids (red) and cGAS (green) in presence of DAPI (blue). Co-localized regions are shown in yellow in the right panels. Scale bar denotes 10 μm. Data are representative of 3 independent experiments.

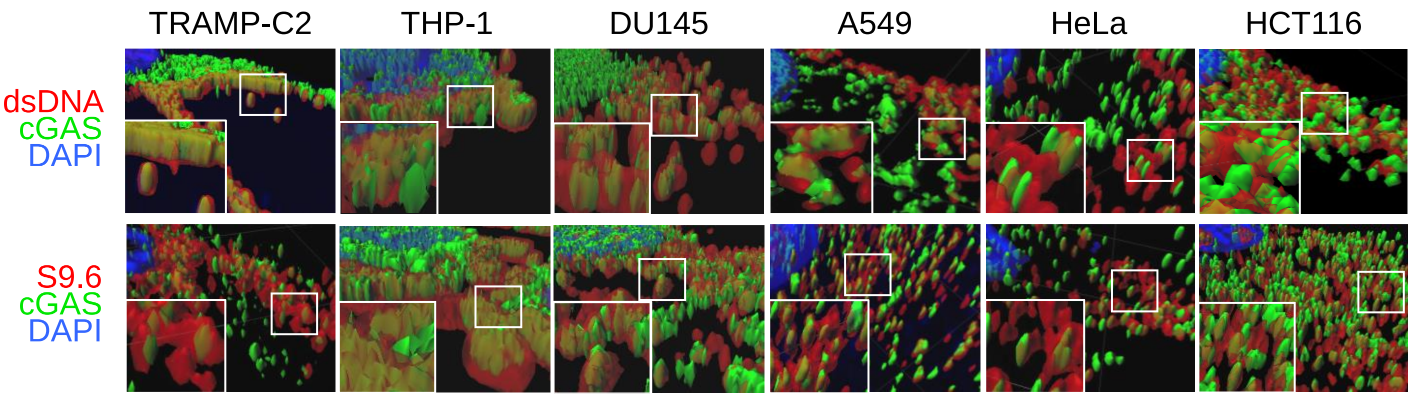

Figure S4. 3D-rendered Confocal Images of Co-staining of Cytosolic DNA and cGAS in Tumor Cells.

3D iso-surface plots of co-staining of cGAS (green) with dsDNA (upper row; red) or RNA:DNA hybrids (lower row; red) described in (Fig. 4A) were generated by Imaris. DAPI was used to counterstain nuclear DNA (blue). White boxes indicate the magnified areas shown in the left lower corner. Z-stack images were acquired by confocal microscopy. Data are representative of 3 independent experiments.

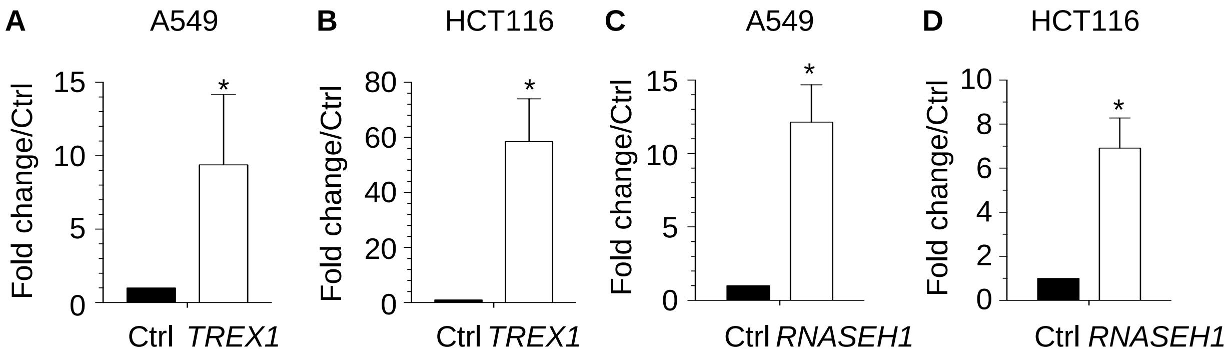

Figure S5. Overexpression of *TREX1 or RNASEH1* in A549 and HCT116 Cells

A and B, *TREX1* transcript levels were determined by real-time PCR in (A) A549 and (B) HCT116 cells described in (Fig. 3B). **C and D**, *RNASEH1* transcript levels were determined by real-time PCR in (C) A549 and (D) HCT116 cells described in (Fig. 3C). All data are presented as mean ± SD of 3 independent experiments.

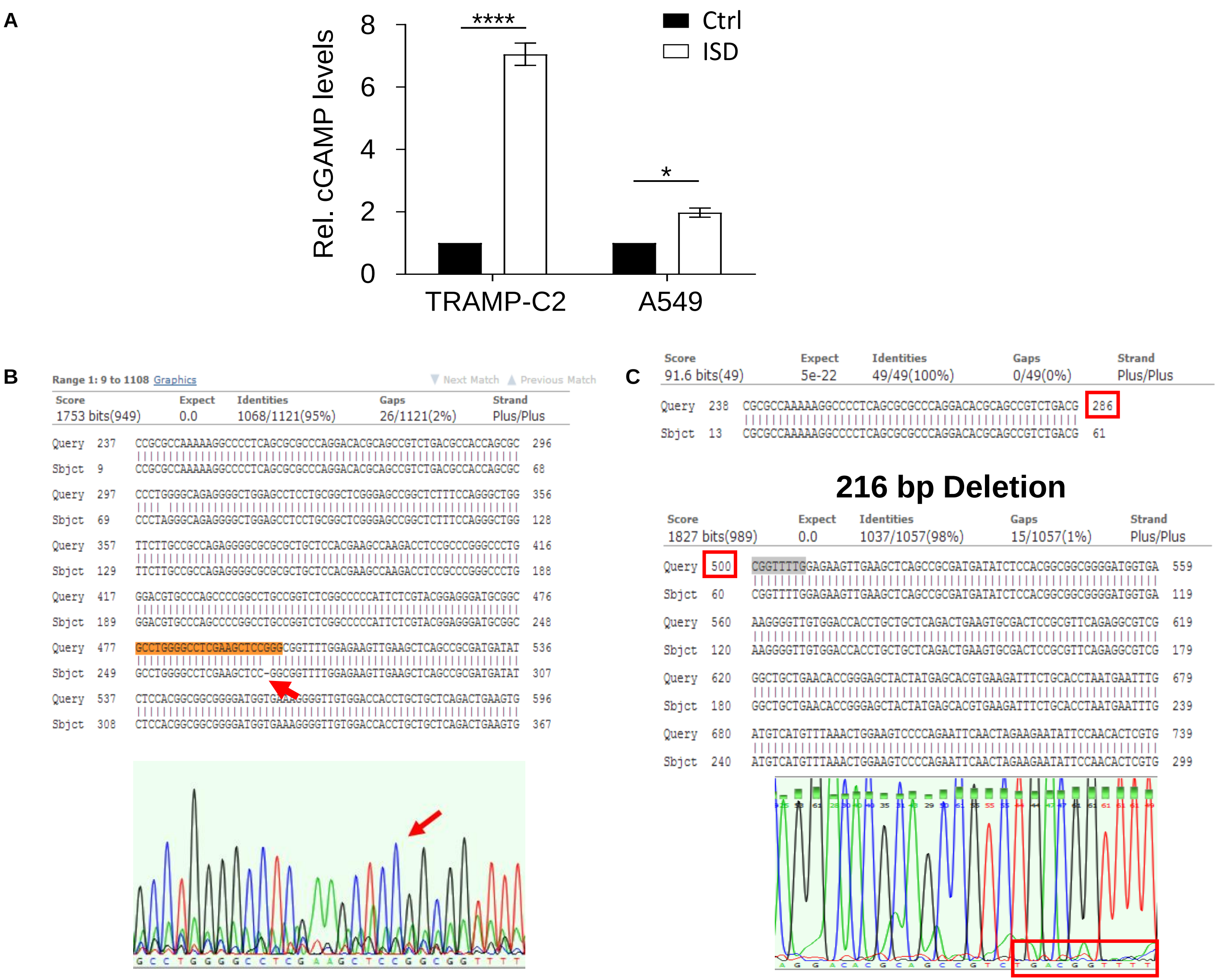

Figure S6. cGAMP levels in TRAMP-C2 and A549 cells upon cGAMP stimulation and generation of cGAS KO cells.

A, TRAMP-C2 and A549 cells were transfected with PBS (ctrl) or 4 µg/ml ISD for 16 hours. Cells were analyzed for the production of cGAMP by ELISA. Expression values were normalized to control cells. **B-C**, Sequencing results of genomic DNA encoding the cGAS gene in (A) A549 and (B) HeLa cells transfected with *cGAS*-specific guide RNAs ($cGAS^{CRISPR}$) post selection. Red arrows (A549) or red boxes (HeLa) indicate the position of mutations or deletions.

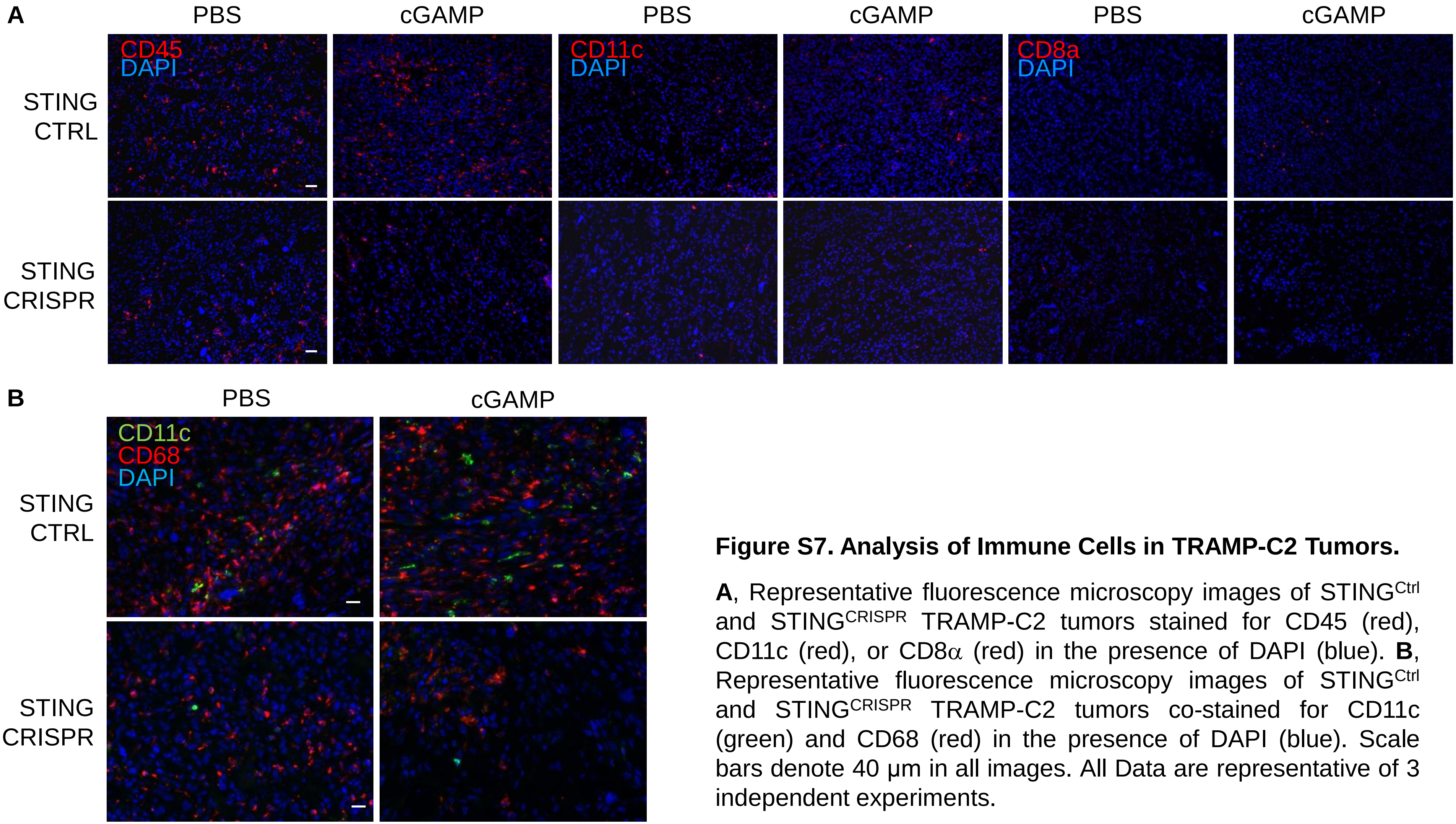

Figure S7. Analysis of Immune Cells in TRAMP-C2 Tumors.

A, Representative fluorescence microscopy images of $STING^{Ctrl}$ and $STING^{CRISPR}$ TRAMP-C2 tumors stained for CD45 (red), CD11c (red), or CD8α (red) in the presence of DAPI (blue). **B**, Representative fluorescence microscopy images of $STING^{Ctrl}$ and $STING^{CRISPR}$ TRAMP-C2 tumors co-stained for CD11c (green) and CD68 (red) in the presence of DAPI (blue). Scale bars denote 40 μm in all images. All Data are representative of 3 independent experiments.

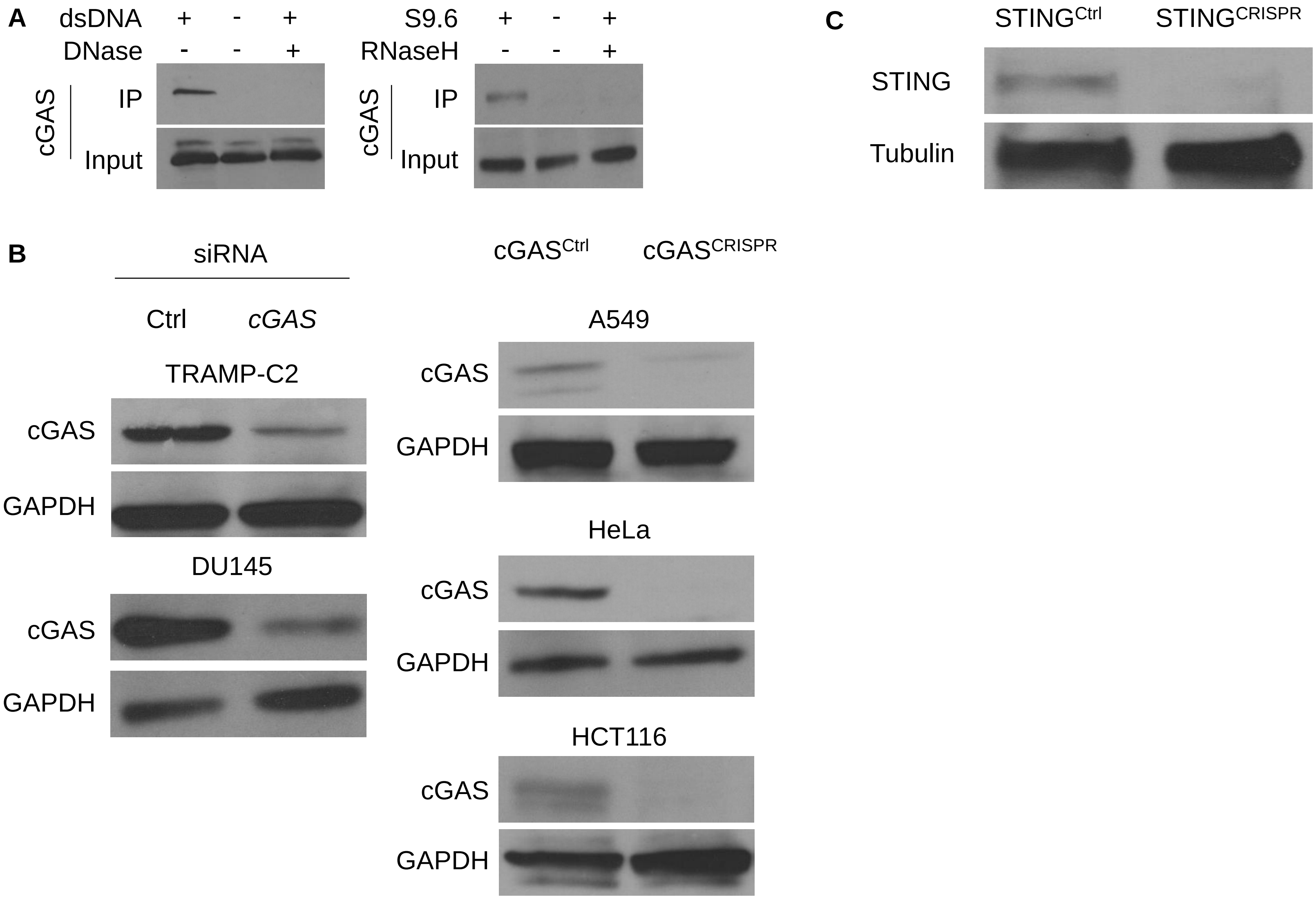

Figure S8. Replicate images for western blots.

A, Replicate images for Fig. 2B. **B**, Replicate images for Fig. 3A. **C**, Replicate images for Fig. 5A.
